# Daurichromenic Acid from the Chinese Traditional Medicinal Plant *Rhododendron dauricum* Inhibits Sphingomyelin Synthase and Aβ Aggregation

**DOI:** 10.3390/molecules25184077

**Published:** 2020-09-07

**Authors:** Hadya Virupaksha Deepak, Mahadeva M. M. Swamy, Yuta Murai, Yoshiko Suga, Masaki Anetai, Takuro Yo, Masahiro Kuragano, Koji Uwai, Kiyotaka Tokuraku, Kenji Monde

**Affiliations:** 1 Graduate School of Life Science, Hokkaido University, Sapporo 001-0021, Japan; deepudarshu@sci.hokudai.ac.jp (H.V.D.); mswamy.madegowda@gmail.com (M.M.M.S.); ymurai@sci.hokudai.ac.jp (Y.M.); 2 Faculty of Advanced Life Science, Hokkaido University, Sapporo 001-0021, Japan; suga@sci.hokudai.ac.jp (Y.S.); glycinoeclepin-a@hb.tp1.jp (M.A.); 3 Department of Applied Sciences, Muroran Institute of Technology, 27-1 Mizumoto-cho, Muroran 050-8585, Japan; udasan_16@yahoo.co.jp (T.Y.); gano@mmm.muroran-it.ac.jp (M.K.); uwai@mmm.muroran-it.ac.jp (K.U.); tokuraku@mmm.muroran-it.ac.jp (K.T.)

**Keywords:** daurichromenic acid, sphingomyelin synthase, amyloid β, dual inhibitor, lipid-related disease

## Abstract

Species of the genus *Rhododendron* have been used in traditional Chinese medicine, with the medicinal herb “Manshanfong” used as an expectorant and for the treatment of acute bronchitis. Daurichromenic acid (DCA), a constituent of *Rhododendron dauricum*, is a meroterpenoid with antibacterial, anti-HIV, and anti-inflammatory activities. However, the mechanisms underlying these pharmacologic activities are poorly understood. To develop new drugs based on DCA, more information is required regarding its interactions with biomolecules. The present study showed that DCA inhibits the activity of the enzyme sphingomyelin synthase, with an IC_50_ of 4 µM. The structure–activity relationships between DCA and sphingomyelin synthase were evaluated using derivatives and cyclized hongoquercin A. In addition, DCA was found to inhibit amyloid β aggregation. These results may help in the design of effective drugs based on DCA.

## 1. Introduction

Traditional medicinal plants are promising sources of naturally occurring drugs, especially of natural products targeting membrane proteins, including membrane receptors and enzymes. Daurichromenic acid (DCA), a meroterpenoid consisting of orsellinic acid and sesquiterpene moieties, was isolated from the leaves of *Rhododendron dauricum*, a plant growing in the wild in northern China, eastern Siberia, and the Japanese island of Hokkaido [1]. This species of *Rhododendron* has been used in traditional Chinese medicine, with the medicinal herb “Manshanfong” used as an expectorant and to treat various diseases, such as acute and chronic bronchitis [2]. DCA has also been reported to have antibacterial activity against Gram-positive bacteria [3], as well as having anti-HIV [4], and anti-inflammatory [5] activity, suggesting that DCA may be a medicinal resource in the development of derivatives that can act as novel drugs to treat these conditions. Although DCA exhibits various pharmacological activities, including the induction of cell death in cultured cells [6], its molecular mechanisms of activity remain unclear. Thus, a deeper understanding of its mechanisms of activity in human (patho)physiology and its potential therapeutic uses is required for new drug discovery and pharmacotherapeutics.

Sphingomyelin synthase (SMS) is a cell membrane-located lipid-metabolizing enzyme, which has been reported to play crucial roles in cell death, proliferation, and migration [7]. SMS knockout mice showed a decrease in plasma concentrations of inflammatory cytokines [8], as well as resistance to the development of high-fat diet-induced obesity [9], insulin resistance [10], Alzheimer’s disease [11], and tumorigenesis [12]. SMS activity was shown to be inhibited by the naturally occurring compounds ginkgolic acid, from the leaves of *Gingko biloba* [13], and malabaricones A–C and E, from the fruits of *M. cinnamomea* [14]. The latter inhibitors may be especially safe, with fewer side effects than other SMS inhibitors, because they were isolated from an edible fruit. Similarly, DCA may directly or indirectly affect SMS activity, regulating cell death and anti-inflammatory activity. Elucidation of the mechanism by which DCA interacts with SMS could contribute to the development of novel therapeutic agents. Moreover, the nontoxic nature of DCA makes it a suitable candidate for further development as a new drug or medicinal supplement.

Modulation of the activity of a specific enzyme or receptor by a small molecule is one of the cornerstones of modern target-based drug discovery. However, small molecules frequently bind to multiple target molecules, influencing both drug efficacy and safety [15,16]. Although the one-drug-hits-one target approach was dominant in the post-genomic era, it is regarded as inadequate to address complex diseases [17]. The multitarget approach has become a fruitful area of drug discovery, particularly in developing medicines to treat major complex diseases. For example, natural products such as genistein [18], curcumin, and tashinone [19] have been reported to inhibit amyloid β (Aβ) and human islet amylin (hIAPP) associated with type 2 diabetes. Another compound, miltirone, isolated from *Salvia miltiorrhiza*, was shown to be a dual inhibitor of P-glycoprotein and cell growth in doxorubicin-resistant HepG2 cells [20]. Furthermore, cannabinoid compounds, which are structurally related to DCA, have been reported to inhibit the formation of Aβ fibrils and aggregates, which have neuronal and microglial-activated neurotoxicity [21].

To determine the biological properties of DCA and its derivatives and whether they are promising candidate drugs, the ability of these compounds to inhibit SMS activity was analyzed, as was the structure–activity relationship of these compounds in cell-based SMS assays. Furthermore, the effects of DCAs and natural SMS inhibitors on Aβ aggregation were assessed by microtiter-scale high-throughput screening (MSHTS) assays.

## 2. Results and Discussion

### 2.1. Preparation of Daurichromenic Acid *(**1**)* and Its Derivatives *(**2**–**7**)*

**Scheme 1:**
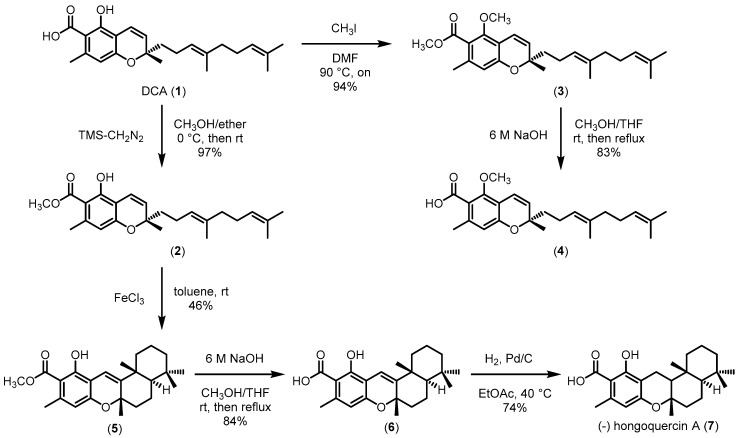
Preparation and synthesis of DCA (**1**) and its derivatives.

The identity of DCA (**1**) isolated from *Rhododendron dauricum* was confirmed by ^1^H- and ^13^C-nuclear magnetic resonance (NMR) and by electrospray ionization-mass spectrometry (ESI-MS) and compared with previously reported values [22]. To understand the structure–activity relationship (SAR) between DCA and its target molecules, DCA derivatives were synthesized. Compound (**2**) was prepared from DCA by selective methyl esterification of its carboxylic acid moiety by TMS-CH_2_N_2_. Compound (**3**) was prepared by protecting the carboxylic acid and hydroxy groups with iodomethane, and compound (**4**) was prepared by hydrolysis of the methyl ester group of compound (**3**). Subsequently, hongoquercin A (**7**), which has been isolated from an unidentified terrestrial fungus [23] and exhibits antibacterial properties toward methicillin-resistant *Staphylococcus aureus* and vancomycin-resistant *Enterococcus faecium* [24,25], was prepared from compound (**2**). In the first step, compound (**2**) was treated with FeCl_3_ to obtain the cyclized derivative compound 5 (**5**). The methyl ester group of (**5**) was hydrolyzed to yield compound (**6**), and the double bond at the benzyl position of compound (**6**) was reduced by Pd/C and H_2_ to obtain hongoquercin A (**7**) in moderate yield (Scheme 1).

### 2.2. SMS Inhibition by DCA and Its Derivatives

**Figure 1:**
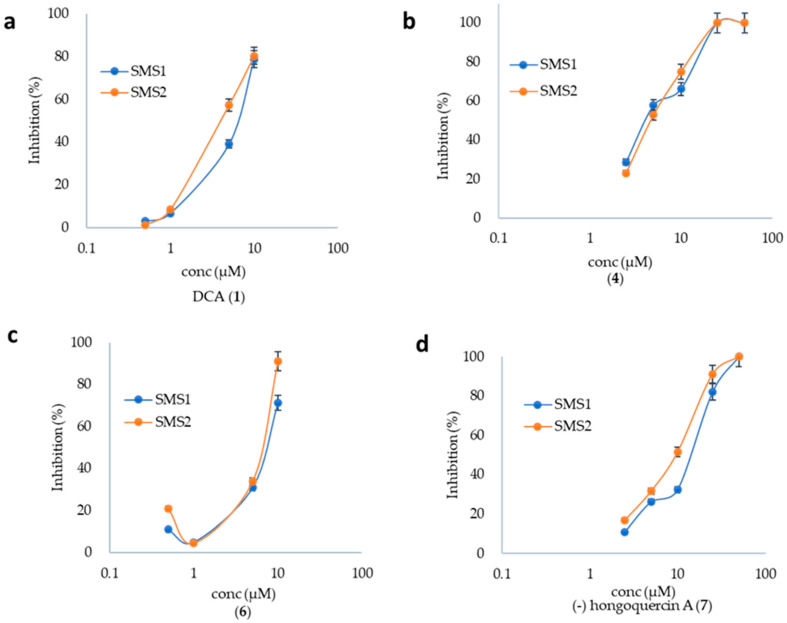
Inhibition of SMS1 and SMS2 activities by (**a**) DCA (**1**), (**b**) derivative (**4**), (**c**) derivative (**6**) and (**d**) derivative (**7**). IC_50_ values were measured using a cell lysate assay. SMS-expressing cell lysates and compounds were incubated for 3 h at 37 °C, and the extracted fluorescent lipids were directly analyzed by HPLC. Each point represents the mean ± SD of triplicate assays.

The ability of DCA and its derivatives to inhibit the SMS isozymes SMS1 and SMS2 was evaluated using a cell lysate assay and the fluorescent substrate C6-NBD (4-nitrobenzo-2-oxa-1,3-diazole)-Cer. DCA (**1**) and compounds **4**, **6**, and **7** showed relatively moderate inhibitory activities, with IC_50_ values of 7, 17, 9, and 4 μM, respectively, for SMS1, and 4, 10, 7, and 5 μM, respectively, for SMS2 (Figure 1), similar to other natural compounds [13,14]. These findings indicated that the methyl esters of DCA derivatives, compounds **2**, **3**, and **5**, were inactive in these assays. Thus, the carboxylic acid group of DCAs are essential moieties for their inhibition of SMSs. However, the SMS has not been succeeded in its crystallization. Therefore, a direct comparison with the previously isolated SMS inhibitor ginkgolic acid [13] has been examined. The chemical similarity of ginkogolic acid to DCA, as it is a long alkyl chain and a carboxylic acid moiety in an aromatic ring, shows that these two inhibitors also have the same functional group participation to inhibit SMS. Further, a previous report [26] concerning the mode of action for Aβ aggregation inhibition for small molecules gave the insight that the crucial role of a hydrophobic-side hydrocarbon chain and an aromatic ring as well as its two hydrophilic functions causes significant interaction with the amyloid.

### 2.3. Inhibition of Aβ Aggregation by DCA and Its Derivatives

**Figure 2:**
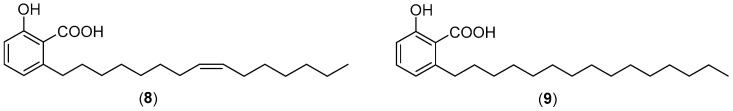
ginkgolic acids (**8**, **9**).

**Figure 3:**
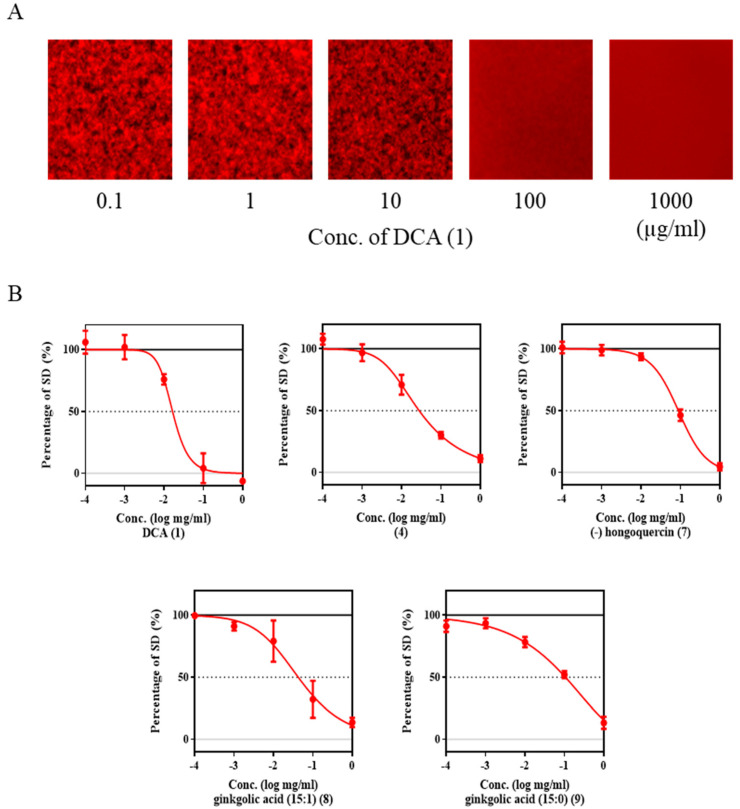
(**A**) Inhibition of Aβ_42_ aggregation by DCA, its derivatives, and natural sphingomyelin synthase inhibitors, as shown by incubation of 30 nM QDAβ and 30 µM Aβ with various concentrations of these compounds. (**B**) Estimation of EC_50_ values from inhibition curves, in which the percent SDs of fluorescence intensities were plotted against the concentrations of these compounds.

Alzheimer’s disease (AD) is a multifactorial disease, which is believed to be caused by complex interactions among several contributing patho-mechanisms. The ability of DCA and its derivatives, along with other natural compounds, ginkgolic acids (**8**, **9**) (Figure 2) and malabaicone A–C (**10**, **11** and **12**), to inhibit Aβ_42_ aggregation was evaluated by microtiter-scale high-throughput screening (MSHTS) assays [27,28]. EC_50_ values were measured from inhibition curves, in which the percent SDs of fluorescence intensities were plotted against the concentration of each compound (Figure 3). Here, 30 nM quantum dot amyloid beta (QDAβ) and 30 µM of Aβ were incubated with different concentrations to evaluate the EC_50_ of all derivatives. DCA and its derivative compound **4** showed relatively potent inhibition activities, with EC_50_ values of 57 and 74 μM, respectively, similar to the EC_50_ of rosmarinic acid (60 μM) [27]. The inhibitory activities of hongoquercin A (**7**) and ginkgolic acids (**8**, **9**) for Aβ aggregation were lower, with EC_50_ values of 150–300 μM. The inhibitory activities of the other cyclized DCAs and malabaricones were lower (Figure S2). These results indicate that DCA, a terpenoid containing a benzopyran moiety, is a candidate inhibitor of Aβ aggregation, and that the carboxylic acid group on aromatics and hydrophobic long alkyl chains may be required for this activity.

### 2.4. DCA and Compound *(**4**)* Inhibit Both SMS and Aβ Aggregation

These studies’ findings indicated that DCA and its derivative compound **4** could be new dual inhibitors of SMS activity and Aβ_42_ aggregation. DCA was recently reported to inhibit the aggregation of Aβ_40_ [29], similar to our findings that DCA inhibits the aggregation of Aβ_42_. Furthermore, SAR studies of the ability of a terpenoid containing a benzopyran moiety to inhibit both SMS activity and Aβ_42_ aggregation suggested that benzoic acid and hydrophobic long alkyl chains are essential for both activities (Table 1).

Natural products are a source of potential pharmacological agents with an abundance of novel chemical entities with various biological activities. The goal of our study is to identify potent SMS inhibitors from a natural source. In this study, we found that DCA (**1**), which is isolated from a *Rhododendron dauricum*, is a potent SMS inhibitor. Leaves of *Rhododendron dauricum* are reported as anti-HIV, used for the treatment of many diseases, and are also involved in cell proliferation, as well as being one of the well-known, practically used Chinese traditional medicines. DCA (**1**) itself possesses several biological activities, popularly anti-HIV and antibacterial, but its molecular mechanisms have not yet been examined. To completely understand the pharmalogical behavior of DCA (**1**) with sphingomyelin synthase enzyme, a series of DCA derivatives **2**–**6** and (−) hongoquercin A (**7**) were synthesized to evaluate functional group participation in the enzyme inhibiton. Structures of isolated and all synthesized compounds were analyzed using various spectroscopic methods such as UV, IR, ESI-MS, and optical rotation for synthesized compounds. Compounds with SMS inhibition properties against cell-based assays and the fluorescent substrate C6-NBD (4-nitrobenzo-2-oxa-1,3-diazole)-Cer share a carboxylic acid moiety which is also related to anti-aggregative properties against amyloid beta protein. MSHTS assays were used to evaluate amyloid beta aggregation activity using DCA and their derivatives. DCA (**1**) and compound **4** with the presence of a carboxylic acid group tend to inhibit both SMS and Aβ aggregation inhibition. Our work, to the best of our knowledge, is the first report on daurichromenic acid and derivatives of DCA and compound **4** as a dual inhibitor for sphingomyelin synthase and amyloid beta aggregation.

## 3. Materials and Methods

NMR spectra were recorded on a Varian Inova instrument (500 MHz) at 25 °C in CDCl_3_, and CD_3_OD using tetramethyl silane (TMS) as an internal standard. The NMR solvents CDCl_3_ and CD_3_OD were purchased from Cambridge Isotope Laboratories (Tewksbury, MA, USA). Chemical shifts (δ) were reported in parts per million (ppm) and coupling constant values (*J*) in Hertz (Hz) relative to CDCl_3_ (1H, δ 7.26; 13C, δ 77.00) or CD_3_OD (1H, δ 3.4, 4.8; 13C, δ 49.3) and tetramethyl silane. The following abbreviations were used for signal multiplicities: s = singlet; d = doublet; t = triplet; q = quartet, and m = multiplet. All other solvents were of reagent grade and were purchased from Kanto Chemical (Tokyo, Japan). ESI-MS spectra were recorded on a JEOL JMS-T100LP spectrometer (JEOL, Akishima, Japan). Melting points were measured on a Micro Melting Point Apparatus (Yanaco Co., Ltd., Kumiyama, Japan). Analytical thin-layer chromatography (TLC) was performed on 0.2 mm silica gel plates (Merck 60 F-254), (Darmstadt, Germany), developed with CHCl_3_:MeOH:H_2_O (65:35:10, lower phase) and sprayed with a mixture of 5% ammonium molybdate and 1% cerium (IV) sulphate in 3.6 N H_2_SO_4_ (Hanessian stain solution), or viewed with a handheld UV lamp (UVGL-58) at 254 nm.

### 3.1. Extraction and Isolation of Active Compounds from Rhododendron dauricum

*Rhododendron dauricum* leaves were collected in Hokkaido, Japan, dried, and ground into powder. The dried powder, weighing 81 g, was extracted three times with 400 mL methanol for 24 h each at room temperature. The methanol extracts were combined and concentrated under reduced pressure, yielding a black residue weighing 24.2 g. The residue was dissolved in 20% MeOH in water (500 mL) and partitioned three times with 200 mL hexane, three times with 200 mL Et_2_O, and three times with 200 mL EtOAc. The solvents were evaporated, and SMS activities were assessed in the hexane, Et_2_O, EtOAc, and water residues. The hexane fraction was more active than the Et_2_O fraction, whereas the EtOAc and water fractions were inactive. The active hexane fraction, weighing 3.8 g, was further fractionated by column chromatography on a spherical silica gel of diameter 40–50 μm (Kanto Silica Gel 60). The active component was identified as DCA, a finding confirmed by ^1^H-NMR and ESI-MS [20]. Yield: 1.53% (1.24 g). Yellow oil; ^1^H NMR (CDCl_3_, 500MHz) δ 11.71 (1H, s), 6.73 (1H, d, *J* = 10 Hz), 6.23 (1H, s), 5.48 (1H, d, *J* = 10 Hz), 5.06–5.12 (2H, m), 2.52 (3H, s), 2.02–2.13 (4H, m), 1.95 (2H, t, 8.3 Hz), 1.68–1.77 (1H, m), 1.67 (3H, s), 1.57–1.59 (6H, 2s), 1.40 (3H, s). ESI–MS (*m*/*z*): 353.46 [M + 1 − 18].

### 3.2. Synthesis of DCA Derivatives

#### 3.2.1. Methyl Ester of DCA (**2**)

A solution of TMS-CH_2_N_2_ in hexane was added to a solution of DCA (compound **1**) (100 mg, 0.270 mmol) in methanol (5 mL) and diethyl ether (5 mL) at 0 °C until the color of the solution became yellow. The reaction mixture was stirred at 0 °C for 0.5 h and at room temperature for 1 h. The reaction was quenched with acetic acid and concentrated under vacuum to yield a residue, which was dissolved in hexane and purified by silica gel column chromatography using hexane/EtOAc (9.5:0.5) as an eluent, resulting in ester **2** [21] at a yield of 97%. Colorless oil; ^1^H NMR (CDCl_3_, 500 MHz) δ 11.97 (1H, s), 6.72 (1H, d, *J* = 10 Hz), 6.18 (1H, s), 5.47 (1H, d, *J* = 10.2 Hz), 5.05–5.11 (2H, m), 3.91 (3H, s), 2.45 (3H, s), 1.93–2.10 (6H, m) 2.97–3.00, 1.57–1.76 (2H, m), 1.66 (3H, s), 1.56–1.58 (6H, 2s), 1.39 (3H, s). ESIMS (*m*/*z*): 385.50 [M + H]^+^.

#### 3.2.2. Methyl(*S*,*E*)-2-(4,8-dimethylnona-3,7-dien-1-yl)-5-methoxy-2,7-dimethyl-2H-chromene-6 carboxylate (**3**)

CH_3_I (55 mg, 0.400 mmol) was added to a solution of DCA (**1**) (59.0 mg, 0.160 mmol) in DMF (3 mL) at room temperature, and the reaction mixture was stirred at 90 °C overnight. The reaction mixture was brought to room temperature and extracted with EtOAc. The organic layer was concentrated, and the residue was dissolved in hexane and purified by silica gel column chromatography using hexane/EtOAc (9:1) as an eluent, resulting in 59.0 mg of compound **3** (yield 94%). Colorless oil; ^1^H NMR (CDCl_3_, 500 MHz) δ 6.55 (1H, d, *J* = 10 Hz), 6.42 (1H, s), 5.56 (1H, d, *J* = 10 Hz), 5.06–5.11(2H, m), 3.89 (3H, s), 3.78 (3H, s), 2.24 (3H, s), 1.94–2.05 (6H, m), 1.56–1.58 (2H, m), 1.67 (3H, s), 1.56–1.58 (6H, 2s), 1.38 (3H, s). ESIMS (*m*/*z*): 399.81 [M + H]^+^.

#### 3.2.3. (*S*,*E*)-2-(4,8-Dimethylnona-3,7-dien-1-yl)-5-methoxy-2,7-dimethyl-2H-chromene-6-carboxylic acid (**4**)

A solution of 6 M NaOH (5 mL) was added to a solution of compound **3** (50.0 mg, 0.130 mmol) in methanol (2 mL) and THF (2 mL) at room temperature, and the reaction mixture was stirred at 100 °C for 2 h. The reaction mixture was brought to room temperature, the solvent was evaporated, and the residue was neutralized with 2 N HCl and extracted with EtOAc. The organic layer was concentrated, and the residue was dissolved in hexane and purified by silica gel column chromatography using hexane/EtOAc (9.5:0.5) as an eluent, resulting in 59.0 mg of compound **4** (yield 83%). Colorless oil; 1H NMR (CDCl_3_, 500 MHz) δ 10.50 (1H, s), 6.55 (1H, d, *J* = 10.2 Hz), 6.53 (1H, s), 5.63 (1H, d, *J* = 9.2 Hz), 5.06–5.11(2H, m), 3.87 (3H, s), 2.50 (3H, s), 1.94–2.15 (6H, m) 2.97–3.00, 1.65–1.80 (2H, m), 1.67 (3H, s), 1.56–1.58 (6H, 2s), 1.41 (3H, s). ESIMS (*m*/*z*): 385.32 [M + H]^+^.

#### 3.2.4. (−)-(5*R*,8*S*,10*R*)-9,15-Didehydro hongoquercin A methyl ester (**5**)

Ferric chloride (81 mg, 499 μmol) was added to a solution of **2** (194 mg, 505 μmol) in toluene (10 mL) at room temperature and the solution stirred for 8 h until the disappearance of the starting material. The toluene was evaporated, and the residue dissolved in 10 mL of water, followed by extraction with ethyl acetate. The combined organic layer was dried over Na_2_SO_4_, and the residue was dissolved in hexane and purified by silica gel column chromatography using hexane/EtOAc (9.5:0.5) as an eluent, resulting in compound **5** as a white solid (yield, 46%). ^1^H NMR (CDCl_3_, 500 MHz) δ 11.99 (1H, s), 6.50 (1H, s), 6.19 (1H, s), 3.91 (3H, s), 2.45 (3H, s), 2.20 (1H, ddd, *J* = 3.5, 3.5, 13.0Hz), 2.05 (1H, d, *J* = 12.5Hz), 1.90 (1H, ddd, *J* = 4.5, 13.0, 13.0Hz), 1.80 (1H, d, *J* = 13.5Hz), 1.65–1.70 (1H, m), 1.58–1.62 (1H, m), 1.44 (3H, s), 1.41–1.52 (3H, m), 1.17 (3H, s), 1.09–1.20 (2H, m), 0.92 (3H, s), 0.86 (3H, s); ESIMS (*m*/*z*): 385.10 [M + H]^+^.

#### 3.2.5. (−)-(5*R*,8*S*,10*R*)-9,15-Didehydro hongoquercin A (**6**)

A solution of 6 M NaOH (1.5 mL) was added to a solution of compound **5** (27.0 mg, 69.9 μmol) in methanol (1 mL) and THF (2 mL). The mixture was refluxed for 2 h, acidified with 2% HCl, and extracted with CH_2_Cl_2_. The combined organic layer was washed with saline solution, dried over Na_2_SO_4_, and concentrated. The crude residue was purified by silica gel column chromatography using hexane/EtOAc (4:1) as an eluent, resulting in compound **6** [20] as a white solid (yield 84%). ^1^H NMR (CDCl_3_, 500 MHz) δ 11.69 (1H, s), 6.50 (1H, s), 6.24 (1H, s), 2.53 (3H, s), 2.20 (1H, ddd, *J* = 3.5, 3.5, 13.0 Hz), 2.05 (1H, d, *J* = 12.5 Hz), 1.90 (1H, ddd, *J* = 4.5, 13.0, 13.0 Hz), 1.79 (1H, d, *J* = 13.5 Hz), 1.64–1.70 (1H, m), 1.56–1.61 (1H, m), 1.42 (3H, s), 1.37–1.51 (3H, m), 1.16 (3H, s), 1.08–1.20 (2H, m), 0.91 (3H, s), 0.86 (3H, s); ESI-MS (*m*/*z*): 393.30 [M + Na]^+^.

#### 3.2.6. (−) Hongoquercin A (**7**)

A solution of 10% Pd/C (0.028 mmol) was added to a solution of **6** (0.140 mmol) in EtOAc (10 mL) while stirring, and the reaction mixture was further stirred overnight at 40 °C under a H_2_ atmosphere. The solid was filtered off and the filtrate was concentrated under a vacuum. The residue was redissolved in hexane and purified by silica gel column chromatography using hexane/EtOAc (4:1) as an eluent, resulting in compound **7** (yield 74%) as a white solid. Its structure was confirmed by ^1^H- and ^13^C-NMR and ESI-MS and compared with previous results [18]. ^1^H NMR (500 MHz, CDCl_3_) δ 11.78 (1H, s), 6.14 (1H, s), 2.63 (1H, dd, *J* = 16.8 Hz), 2.44 (3H, s), 2.19–2.25 (m, 1H), 2.01 (1H, dt, 3.1, 13.0 Hz), 1.69 (2H, m), 1.54–1.60 (3H, m), 1.48 (1H, dd, *J* = 5.4, 13.0 Hz), 1.38–1.43 (1H, m), 1.28–1.35 (2H, m), 1.13 (3H, s), 0.96 (1H, dd, *J* = 2.2, 12.4 Hz), 0.90 (1H, dd, *J* = 3.2, 13.0 Hz), 0.85 (3H, s), 0.83 (3H, s), 0.78 (3H, s) [α${]}_{D}^{23}$ = −109.1 (c 0.10, CHCl_3_) ESIMS (*m*/*z*): 372.52 [M + H]^+^.

### 3.3. SMS Assay

ZS/SMS1 and ZS/SMS2 cells (protein concentration 0.1 µg/µL) were diluted in 20 mM Tris-buffer (pH 7.5) and sonicated. A 1 µL aliquot of each compound at the desired concentration was added to 100 µL aliquots of the cell lysates and the solutions were incubated at 37 °C for 30 min. A 1 µL aliquot of C6-NBD-ceramide was added to each solution and the solutions were incubated for 3 h at 37 °C. The reactions were stopped by addition of 400 µL of MeOH/CHCl_3_ [1/2 (*v/v*)], and the mixtures were shaken and centrifuged at 1500 rpm for 5 min. The formation of C6-NBD-sphingomyelin was quantified by HPLC determination of its peak area. Inhibitory activity was quantified by a reverse-phase HPLC assay using a JACSO HPLC system, equipped with a PU-2089 Plus and FP-2020 Plus set at λex = 470 nm and λem = 530 nm. A 50 × 4.6 YMC-Pack Diol-120-NP column (5-µm particle size) was used with the mobile phase (IPA/hexane/water) at a flow rate of 1.0 mL/min

### 3.4. Aβ Inhibition Aggregation Assay

The aggregation inhibitory activities of plant extracts, fractions, DCA, DCA derivatives, and naturally occurring SMS inhibitors (compounds **8**, **9**, and **10**) were measured using a modified MSHTS system [1,25]. Various concentrations of each were incubated with 30 nM QD-labeled Aβ_40_ (QDAβ), and 30 μM Aβ_42_ in PBS containing 5% EtOH and 3% DMSO at 37 °C for 24 h in 1536-well plates (782096, Greiner) (Kremsmünster, Austria). The center of each well as viewed with an inverted fluorescence microscope (TE2000, Nikon)) (Tokyo, Japan) equipped with a color CCD camera (DP72, Olympus) (Tokyo, Japan), and the central region of each well were measured with ImageJ software Ver 1.53b (NIH) to determine the standard deviations (SD) of fluorescence intensities of 40,000 pixels (200 × 200 pixels: 320 × 320 μm) [29,30]. The SD values, which were approximately proportional to the amount of aggregates, were plotted against the concentrations of the putative inhibitors to generate inhibition curves, and the EC_50_ values were determined.

## 4. Conclusions

The present study showed that DCA, a terpenoid from the leaves of *Rhododendron dauricum*, is a natural inhibitor of sphingomyelin synthase. Several of its derivatives, including compounds **4**, **6**, and **7**, also showed good inhibitory activity against SMS. In addition, DCA and compound **4** were found to inhibit Aβ_42_ aggregation. These properties suggest that DCA is a suitable candidate for further development as a new drug or medicinal supplement to treat diseases of abnormal lipid metabolism and to prevent Alzheimer’s disease.

## Figures and Tables

**Table 1:** Inhibitory activities of DCA, its derivatives, and natural sphingomyelin synthase towards SMS activity and Aβ_42_ aggregation.

Compounds	SMS Assay (SMS1)	SMS Assay (SMS2)	MHSTS Assay
	(IC_50_ in µM)	(IC_50_ in µM)	(EC_50_ in µM)
**1**	7	4	57
**4**	17	10	74
**7**	4	5	238
**8**	1.5	1.5	149
**9**	2	2	296

## Supplementary Materials

The following are available online. Figure S1: NMR spectra of daurichromenic acid and its derivatives, Figure S2: Aβ aggregation inhibition activity of DCA and its derivatives.
