# Supplementary material for: Daurichromenic Acid from the Chinese Traditional Medicinal Plant Rhododendron dauricum Inhibits Sphingomyelin Synthase and Aβ Aggregation
Source: Molecules. 2020 Sep 7;25(18):4077. doi: 10.3390/molecules25184077 (PMC7571127; doi:10.3390/molecules25184077)
Supplement: Supplementary file 1 [file molecules-25-04077-s001.pdf]

Supplementary data

Daurichromenic acid from the Chinese traditional medicinal plant Rhododendron dauricum inhibits sphingomyelin synthase and Aβ aggregation

Hadya Virupaksha Deepak[1], Mahadeva M. M. Swamy[1], Yuta Murai[1,2], Yoshiko Suga[2], Masaki Anetai[2], Takuro Yo[3], Masahiro Kuragano[3], Koji Uwai[3], Kiyotaka Tokuraku[3] and Kenji Monde[1,2] *

[1]*Graduate School of Life Science, Hokkaido University, Sapporo, Japan, 001-0021*
[2]*Faculty of Advanced Life Science, Hokkaido University, Sapporo, Japan, 001-0021*
[3]*Department of Applied sciences, Muroran Institute of Technology, 27-1 Mizumoto-cho, Muroran, Japan 050-8585,*
*Correspondence: kmonde@sci.hokudai.ac.jp

S-1

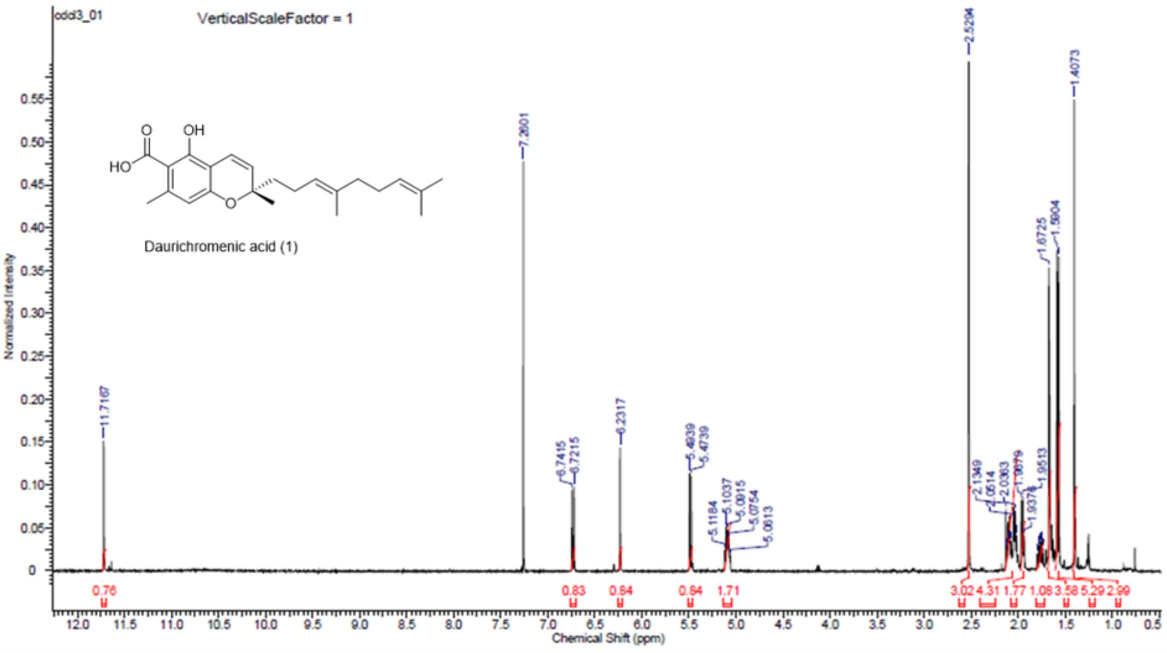

^{1}HNMR of *Daurichromenic acid (DCA)* ***(1)***

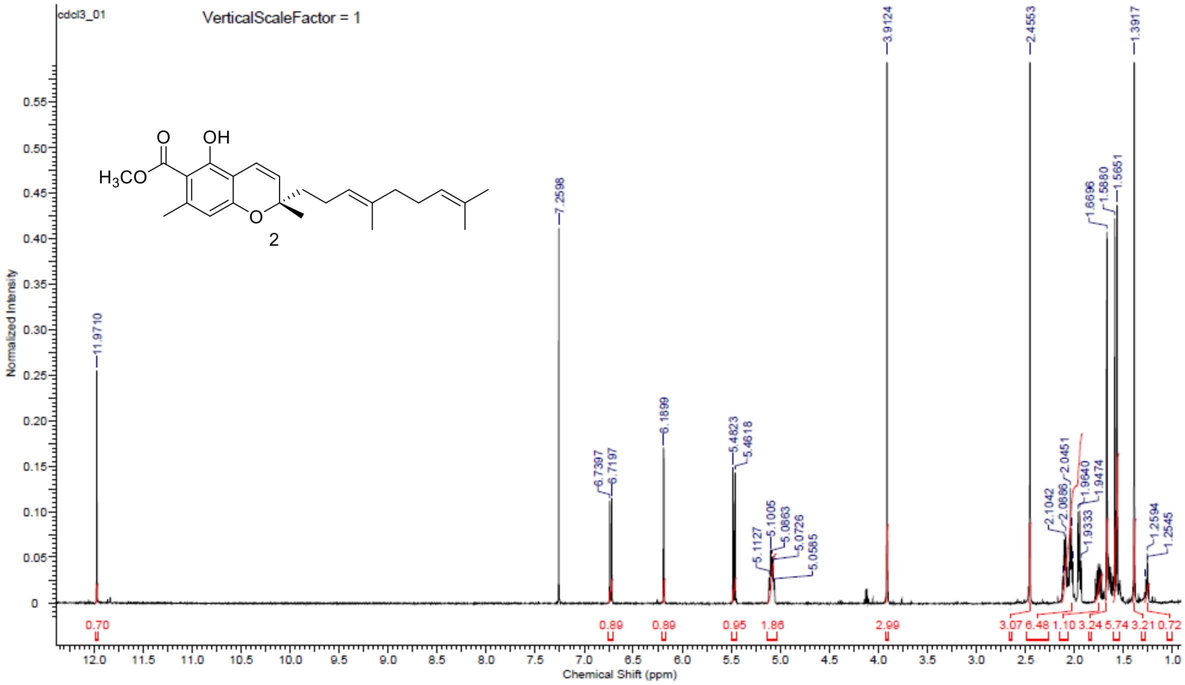

^{1}HNMR *Methyl ester of daurichromenic acid* (**2**)

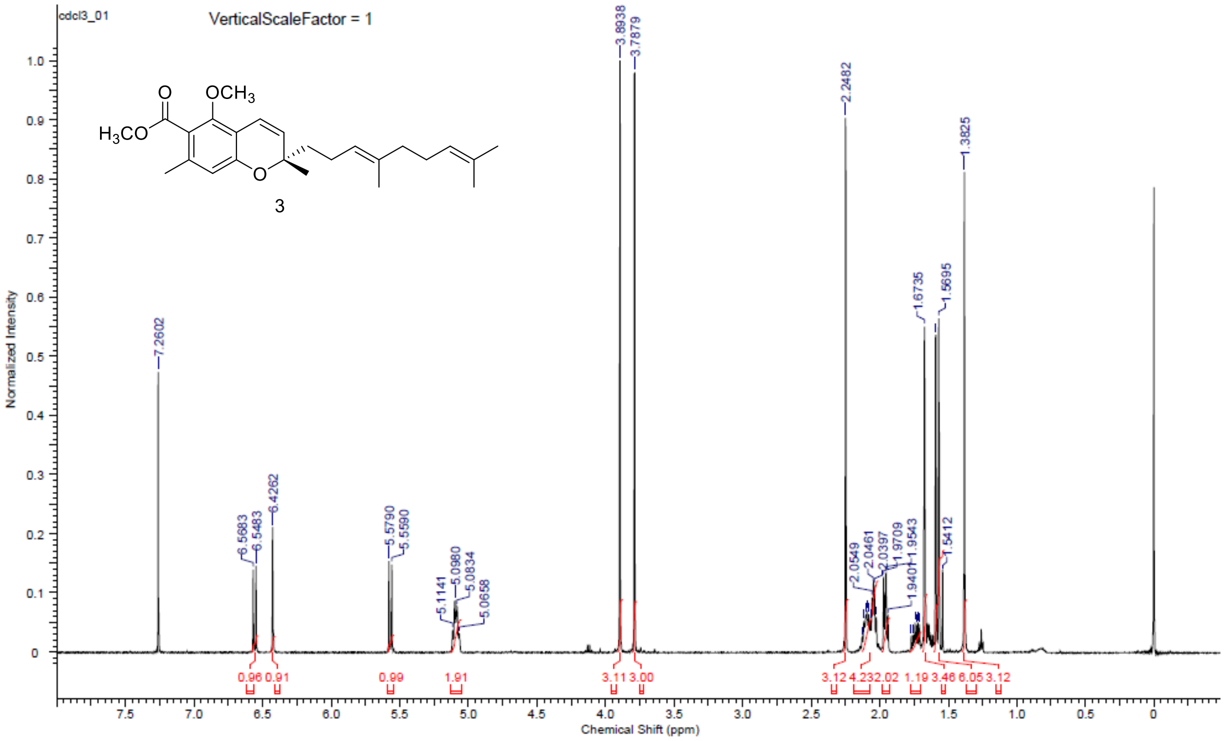

^{1}HNMR of *Methyl(S,E)-2-(4,8-dimethylnona-3,7-dien-1-yl)-5-methoxy-2,7-dimethyl-2H-chromene-6 carboxylate* (**3**)

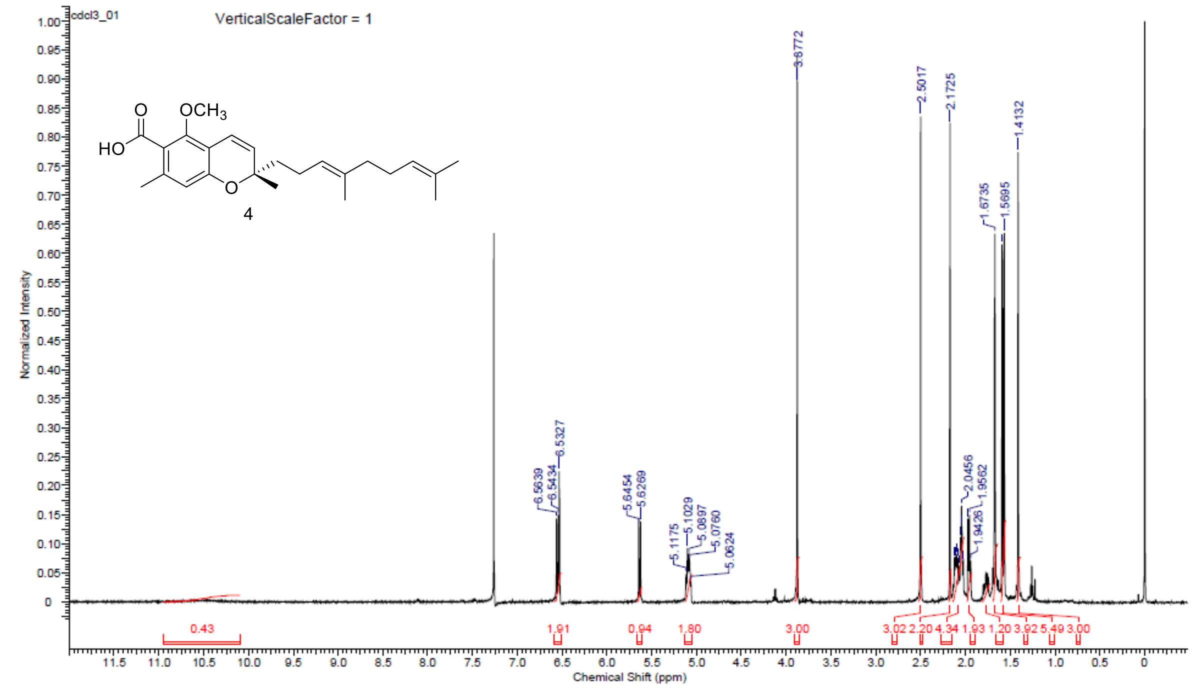

^{1}HNMR of *(S,E)-2-(4,8-dimethylnona-3,7-dien-1-yl)-5-methoxy-2,7-dimethyl-2H-chromene-6-carboxylic acid* (***4***)

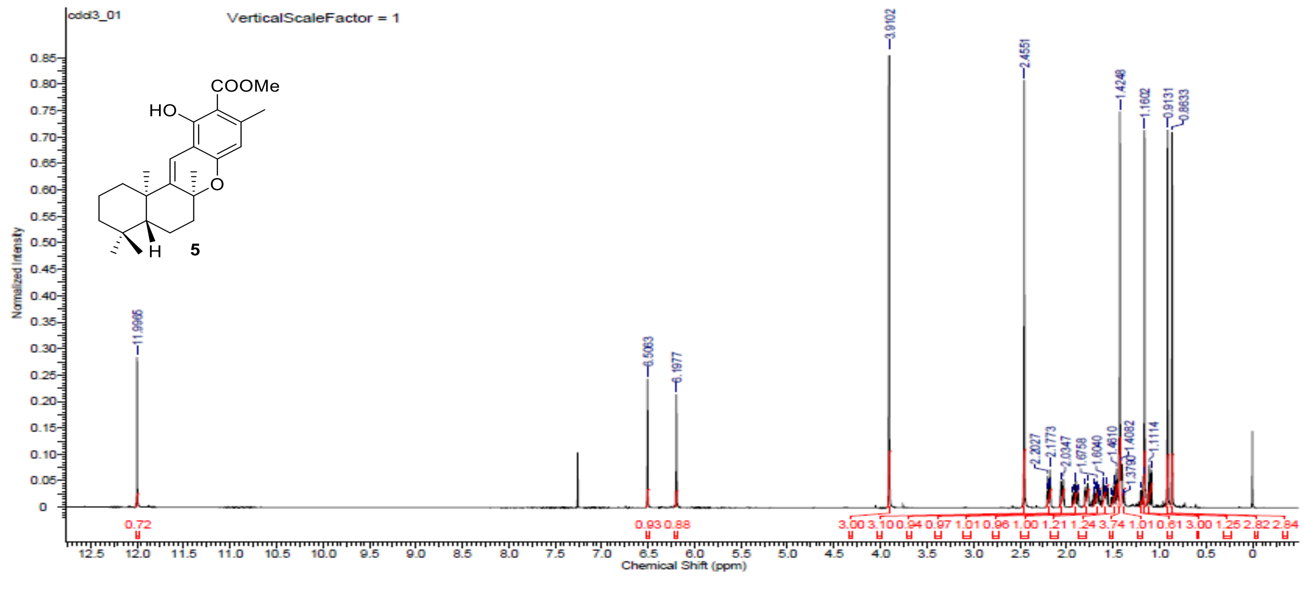

^{1}HNMR of *(–) -(5R,8S,10R)-9,15-didehydro Hongoquercin A methyl ester* (**5**)

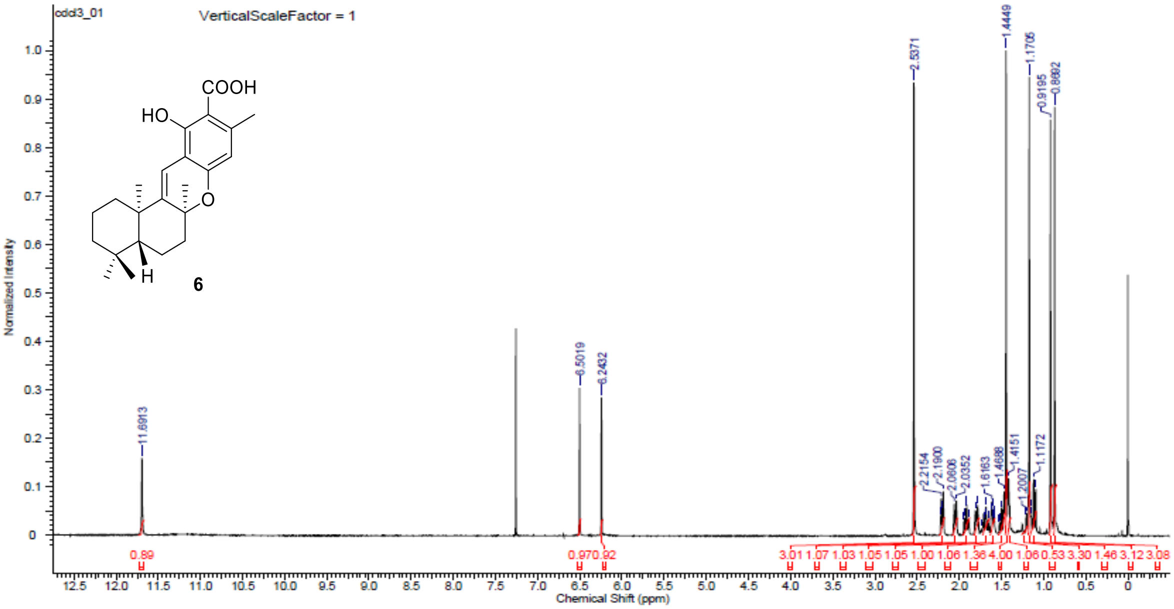

^{1}HNMR of *(–)-(5R,8S,10R)-9,15-didehydro Hongoquercin A* ***(6)***

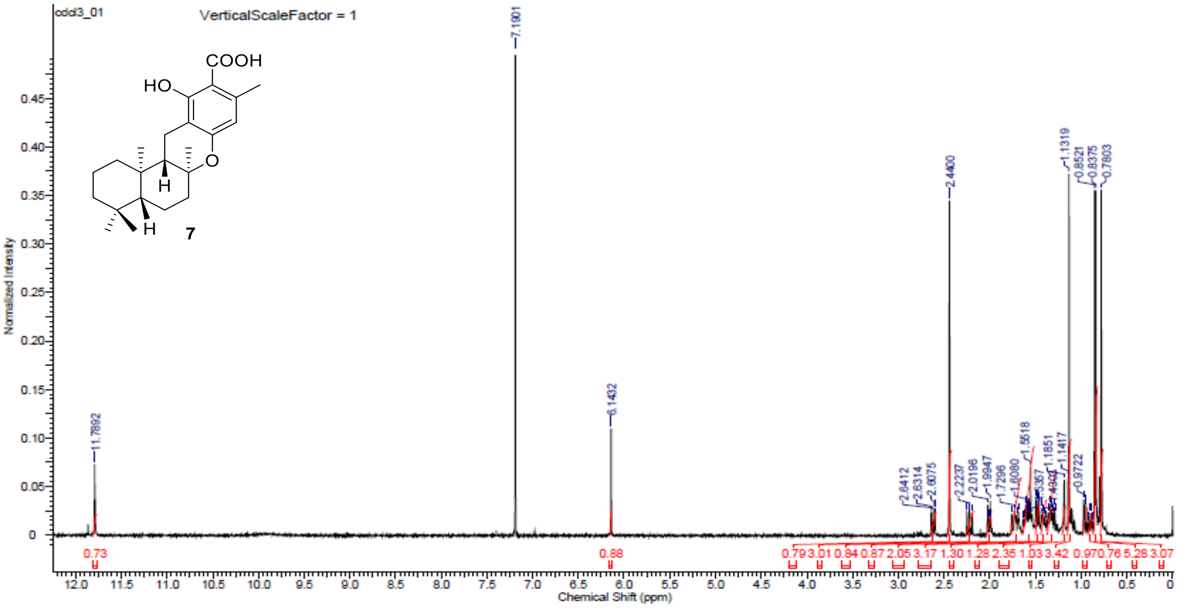

^{1}HNMR of *hongoquercin A* ***(7)***

S-2 Amyloid beta aggregation assay

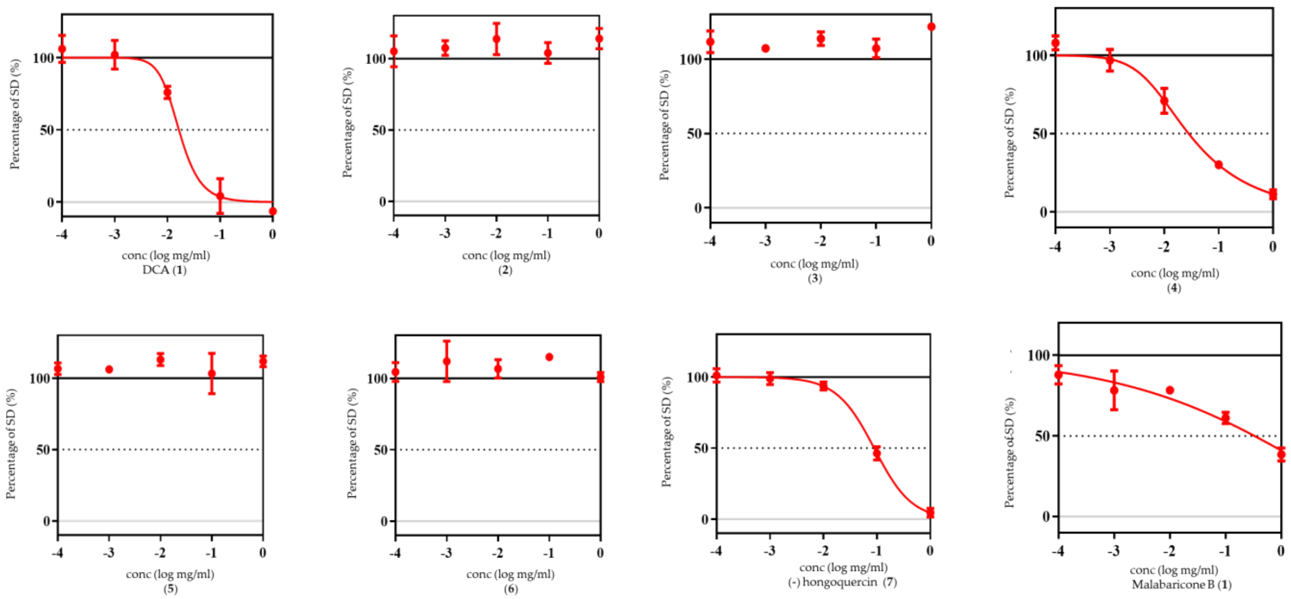

Figure S-2: Amyloid beta aggregation inhibition by daurichromenic acid and its derivatives: Inhibition of Aβ aggregation by daurichromenic acid and its derivatives 30 nM QDAβ and 30 μM Aβ were incubated with various concentrations of daurichromenic acid and its derivatives. Estimation of EC_{50} values from inhibition curve that are plotted percentage of SD of fluorescence intensities in the micrograph versus concentration of daurichromenic acid and its derivatives. Estimation of EC_{50} values from inhibition curve that are plotted percentage of SD of fluorescence intensities in the micrograph versus concentration of daurichromenic acid and its derivatives.
